# Analysis of the clinical factors affecting excellent response of Iodine-131 treatment for pulmonary metastases from differentiated thyroid cancer

**DOI:** 10.1016/j.heliyon.2023.e20853

**Published:** 2023-10-13

**Authors:** Xin-Yu Wu, Bo Li, Jie Zhang, Li-Li Duan, Bing-Xin Hu, Yong-Ju Gao

**Affiliations:** a Department of Nuclear Medicine, Henan Provincial People's Hospital & Zhengzhou University People's Hospital, Zhengzhou, People's Republic of China; b Henan Key Laboratory of Novel Molecular Probes and Clinical Translation in Nuclear Medicine, Zhengzhou, People's Republic of China

**Keywords:** Differentiated thyroid cancer, Iodine radioisotopes, Radiotherapy, Pulmonary metastasis, Excellent response

## Abstract

**Background:**

Iodiene-131 (^131^I) treatment is the primary therapeutic approach for imaging ^131^I-avid pulmonary metastases. The response to radioiodine (RAI) treatment is an important prognostic factor in patients with pulmonary metastases from differentiated thyroid cancer (DTC). Patients who achieve an excellent response (ER) to ^131^I treatment show significantly reduced disease-related mortality. This study aimed to retrospectively analyse the clinical data and therapeutic effects of ^131^I treatment in patients with DTC and pulmonary metastases and to screen out the clinical factors affecting ER.

**Materials and methods:**

The study included a total of 75 patients with exclusively Iodine-131 avid (^131^I-avid) pulmonary metastases who underwent ^131^I treatment. Relevant clinical data for these patients were collected. Following treatment, the status of DTC metastatic lesions was categorized as follows: excellent response (ER), biochemical incomplete response (BIR), structural incomplete response (SIR), or indeterminate response (IDR). Gender, age at diagnosis, pathological type, stages (TNM), stimulated thyroglobulin (sTg) value before initial ^131^I treatment, metastatic nodule size, and type of post-treatment whole body scan (Rx-WBS) were recorded. Mono-factor analysis and binary logistic regression analyses were used to identify the factors that might affect the ER in DTC pulmonary metastases. The receiver operating characteristic (ROC) curve of the sTg value was used to predict the ER of ^131^I treatment.

**Results:**

All 75 patients with exclusively ^131^I-avid pulmonary metastases received ^131^I treatment and underwent follow-up. Out of the 75 patients, 26 achieved ER, resulting in an excellent response rate of 34.7 % (26/75). Among them, 25 (25/26, 96.2 %) achieved an ER after undergoing two rounds of ^131^I treatment. Binary logistic regression analysis showed that the factors influencing DTC pulmonary metastases excellent response were lower sTg levels [odds ratio (*OR*) = 0.998, *P* < 0.001], micronodular metastases (*OR* = 0.349, *P* = 0.001) and focal distribution on Rx-WBS imaging (*OR* = 0.113, *P* = 0.001). The area under the ROC curve for sTg value predicting ER was 0.876, and the cut-off value was 26.84 ng/mL, with a sensitivity and specificity of 87.9 % and 80.3 %, respectively.

**Conclusions:**

^131^I treatment is effective for ^131^I-avid pulmonary metastases of DTC. Some patients who underwent ^131^I treatment achieved ER. Most patients with ER were obtained after two rounds of ^131^I treatments. Patients with sTg values before initial ^131^I treatment lower than 26.84 ng/mL, micronodular metastases, and focal distribution on Rx-WBS imaging were more likely to achieve ER.

## 1 Introduction

The incidence of distant metastasis in differentiated thyroid cancer (DTC) is 5–25 % [[1], [2], [3], [4], [5], [6]], with the lungs being the most frequent site of distant metastasis. Based on their iodine-concentrating capacity, pulmonary metastases are classified as ^131^I-avid or non-^131^I-avid. Iodiene-131 (^131^I) treatment is the primary therapeutic strategy for ^131^I-avid pulmonary metastases. This treatment improves the disease-specific survival time and disease-free survival rate.

The post-treatment status of DTC metastatic lesions was classified based on the response to therapy classification (RTC) in the 2015 American Thyroid Association (ATA) guidelines as follows: 1) excellent response (ER); 2) biochemical incomplete response (BIR); 3) structural incomplete response (SIR); and 4) indeterminate response (IDR) [7]. Some studies have reported that disease-related mortality rates are approximately 50 % for patients with SIR and <1 % for those with ER. Studies have also demonstrated that 20 % of BIR and 15–20 % of patients with IDR can progress to SIR [8,9]. Therefore, it is important for patients to achieve ER, which significantly affects their overall prognosis. An excellent response to therapy should lead to a decrease in the intensity and frequency of follow-up and the degree of TSH suppression. Few previous studies have discussed the clinically relevant factors influencing ER in patients with exclusively ^131^I-avid pulmonary metastases who received ^131^I treatment.

We retrospectively analyzed data from a cohort of patients with exclusively ^131^I-avid pulmonary metastases from DTC at the same institution to assess the therapeutic effects of ^131^I treatment and to screen for the clinically relevant factors influencing ER.

## 2 Materials and methods

### 2.1 Patients

Patients with DTC who underwent a total or near-total thyroidectomy and cervical lymph node dissection and were treated with ^131^I were retrospectively analyzed from January 2010 to June 2021 at Henan Provincial People's Hospital. The final cohort consisted of 75 patients with exclusively ^131^I-avid pulmonary metastases, with an average age of 47.6 ± 13.5 years (range:14–73 years) at the time of metastases diagnosis. Moreover, 69 (92 %) patients underwent total thyroidectomy, 6 underwent near-total thyroidectomy, and 70 (93.3 %) underwent lymph node dissection.

The diagnosis of patients with DTC with exclusively ^131^I-avid pulmonary metastases was based on clinical and chest CT findings, diagnostic or therapeutic ^131^I whole-body scans and serum Tg values. A patient satisfying the following criteria was considered to have exclusively ^131^I-avid pulmonary metastases: 1) Post-treatment whole body scan (Rx-WBS) and SPECT/CT after initial ^131^I treatment indicated pulmonary metastases (excluding non-specific concentrations such as inflammation, and the abnormal radioactive concentration in the lung above the background was recognised as metastatic lesions); 2) No extrapulmonary distant metastases were identified.

Serum thyroglobulin (Tg) value may be affected by the residual thyroid tissue and anti-Tg antibody (TgAb) levels. Patients in the study were also met the following criteria: 3) Neck ultrasonography (US) showed no residual thyroid tissue or abnormal lymph nodes before initial ^131^I treatment; 4) The results of the ^131^I thyroid uptake test showed a^131^I uptake rate of <5 % at the 24th hour; and 5) TgAb values were within the normal range. This study complied with the principles of the Declaration of Helsinki. It was reviewed and approved by the medical ethics committee of Henan provincial People's Hospital. All patients provided written informed consent for the anonymised publication of their data.

### 2.2 ^131^I treatment and thyroxine suppression therapy

1) Preparation for ^131^I treatment: All patients were required to consume a low-iodine diet and withdraw from the levothyroxine treatment for 4 weeks. Free triiodothyronine (FT3), free thyroxine (FT4), thyroid-stimulating hormone (TSH), Tg, TgAb, neck ultrasonography and chest computed tomography (CT) were performed prior to ^131^I administration. FT3, FT4, TSH, and Tg levels were determined using a Roche Cobas E602 Automatic electrochemiluminescence immunoanalyser from Roche Diagnostics Products (Shanghai) Co., Ltd.; and the Tg value detection range (0.04–500 ng/mL) exceeded the upper limit of dilution detection. TgAb levels were determined using the UniCel DxI 800 (Beckman Coulter USA). Chest CT: The area from the apex to the bottom of the lung was scanned with a reconstruction layer thickness of 5 mm. Scanning parameters: 100–120 KV, and automatic tube current of (100–300 mA). According to chest CT findings, patients were divided into the following categories:ⅰ) Patients with micronodular metastases, defined radiologically as <1 cm in the maximum diameter of the pulmonary metastases (including patients with negative chest CT findings but ^131^I uptake on ^131^I-WBS). ⅱ) Patients with macronodular metastases, defined radiologically as ≥1 cm in the maximum diameter of the pulmonary metastases.
2) Activity of ^131^I administered: Previous investigations have demonstrated that the likelihood of identifying either locoregional or distant metastases on the post-therapy scans increases as either the suppressed or stimulated Tg values rise above 5–10 ng/mL [[10], [11], [12], [13]]. Therefore, postoperative Tg values ≥ 10 ng/mL will lead to additional therapy. Patients in ATA intermediate risk, and sTg ≥10 ng/mL received RAI adjuvant therapy, ^131^I were administered at an activity of 3.70–5.55 GBq; sTg <10 ng/mL for 3.70 GBq. Patients in ATA high risk received RAI therapy. ^131^I were administered at an activity of 5.55 GBq. Pathological results confirmed or chest CT indicated pulmonary metastases for 5.55–7.40 GBq.
3) Rx-WBS imaging and SPECT-CT imaging were performed on day 5 after treatment using Infinia^vc^ hawkeye4 or Discovery NM/CT670 CE Medical Systems, (Milwaukee, WI, USA). The scan parameters were as follows: peak energy at 364 keV with a 20 % energy window and parallel-hole high-energy collimator. Based on the radioactivity distribution observed in the Rx-WBS for pulmonary metastases, the Rx-WBS types were categorized as either focal or diffuse.
4) Thyroxine suppression therapy: Levothyroxine (LT_4_) was given at 48 h after ^131^I administration. Patients with pulmonary metastases were classified as ATA high risk, and TSH values were required to be below 0.1mU/L. Retrospective and prospective studies have demonstrated that TSH suppression to below o.1mU/L may improve outcomes in high risk thyroid cancer patients [14,15].

### 2.3 Evaluation of therapeutic efficacy

The efficacy of ^131^I treatment was evaluated using Rx-WBS and SPECT/CT imaging, Tg values in the TSH-suppressed and TSH-stimulated states, and chest CT. Evaluation criteria: For patients with macronodular metastases, the Response Evaluation Criteria in Solid Tumours (RECIST, version1.1) [16] was used to dynamically evaluate the efficacy. 1) Complete response (CR), disappearance of all lesions; 2) partial response (PR), ≥30 % decrease in the maximum diameter of pulmonary metastases, taking as reference the baseline diameters; 3) progressive disease (PD), ≥20 % increase in the maximum diameter of pulmonary metastases, taking as reference the smallest diameters on study and the maximum diameter must also demonstrate an absolute increase of at least 5 mm, the appearance of one or more new lesions is also considered progression; 4) stable disease (SD), neither sufficient shrinkage to qualify for PR nor sufficient increase to qualify for PD, taking as reference the smallest diameters while on study. For patients with micronodular metastases, the response Evaluation was evaluated based on the serum Tg change (ΔTg%). The efficacy was divided into clinical complete response (either Suppressed Tg < 0.2 ng/mL or TSH-stimulated Tg < 1 ng/mL), partial remission (Suppressed Tg values decreased by 25 % or more, ΔTg%≥ 25 %), stable disease (−25 %<ΔTg% <25 %), and disease progression (ΔTg% <-25 %). Patients with ^131^I-avid pulmonary metastases were treated with RAI therapy and treatments were repeated when objective benefits were demonstrated (decrease the size of the metastatic lesions or a decreasing Tg value). Images were reviewed by two experienced nuclear medicine physicians, and where necessary, radiologists were invited to reevaluate the images.

Post-treatment status of DTC metastatic lesions are classified based on the response to therapy classification (RTC) in the 2015 ATA guidelines as follows: ER: Negative imaging and either TSH-Suppressed Tg < 0.2 ng/mL or TSH-stimulated Tg < 1 ng/mL; 2) BIR: Negative imaging and Suppressed Tg ≥ 1 ng/mL or Stimulated Tg ≥ 10 ng/mL; 3) SIR: Structural or functional evidence of disease with any Tg level; 4) IDR: Nonspecific findings on imaging studies; Suppressed Tg detectable, but <1 ng/mL; Stimulated Tg detectable, but<10 ng/mL.

### 2.4 Statistical analysis

Statistical analysis was performed using SPSS version 18.0. Quantitative data consistent with a normal distribution are expressed as $\bar{x}$ ± s, and non-normally distributed data are expressed as M (P25, P75). A two-sample *t*-test was used to compare normally distributed data. Comparisons of categorical variable rates were compared using the χ 2 test (Fisher's test), and the Mann-Whitney U rank-sum test was used for non-normally distributed data. Finally, Binary Logistic regression was used for multivariate analysis. For univariate analysis, a P value < 0.10 was used as standard enrollment regression analysis. Statistical significance was set at P < 0.05 in the multivariate regression analysis.

## 3 Results

### 3.1 Characteristics of patients with ^131^I-avid DTC pulmonary metastases

There were 75 enrolled patients with a predominance of females (21 males and 54 females). The mean age at diagnosis was 47.6 ± 13.5 years (range: 14–73 years). Out of the 75 patients, 63 (84 %) had papillary thyroid carcinoma, and 12 (16 %) had follicular thyroid carcinoma. Staging of patients was performed according to the American Joint Committee on Cancer (AJCC) 8th edition/TNM classification system for DTC, 61 patients were classified as stageⅠ+Ⅱ, and 14 patients were in stage Ⅲ+Ⅳ. According to the 2015 ATA risk stratification system, 6(8 %) patients were classified as ATA intermediate risk, and 69(92 %) patients were classified as ATA high risk before initial ^131^I treatment. Sixty-eight patients showed faint uptake in the thyroid bed outside the pulmonary metastases on Rx-WBS and SPECT/CT after initial ^131^I treatment. In the R_X-_WBS imaging type, 20 (26.7 %) patients had diffuse distribution and 55 (73.3 %) had focal distribution. Nineteen (25.3 %) patients had macronodular metastases, and 56 (74.7 %) had micronodular metastases (including 7 patients with negative chest CT findings but ^131^I uptake on ^131^I-WBS).

### 3.2 Post-treatment status and cumulative activity

**Fig. 1:**
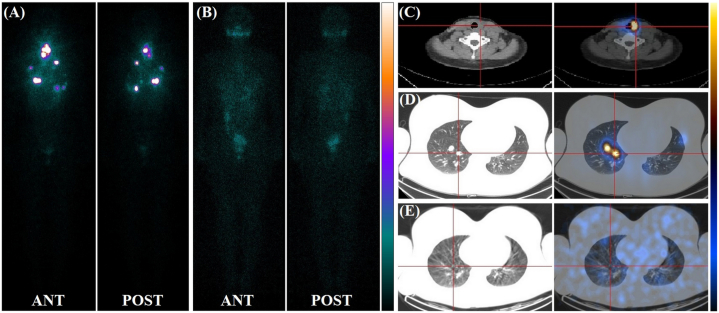
Iodine-131 treatment for DTC pulmonary metastases achieved ER (the Rx-WBS imaging showed focal distribution). Picture specification: A 17-year-old girl had follicular carcinoma of the left lobe of the thyroid with lymph node metastases in area VI (2/4). Chest CT: The maximum diameter of the pulmonary metastases was 1.73 cm. Received twice ^131^I treatments at a activity of 5.55GBq/time. Whole body anterior (ANT) and posterior (POST) plane imaging and SPECT/CT imaging after ^131^I treatment. Imaging after initial ^131^I treatment (A,C,D): Faint ^131^I uptake in the thyroid bed and pulmonary metastases showing focal ^131^I uptake. Imaging after the second ^131^I treatment (B,E): The residual thyroid gland and pulmonary metastases were completely cleared; TgAb negative; sTg value before initial ^131^I treatment was 3314 ng/mL, TSH-suppressed Tg value was＜0.04 ng/mL after the 2nd ^131^I treatment.

**Fig. 2:**
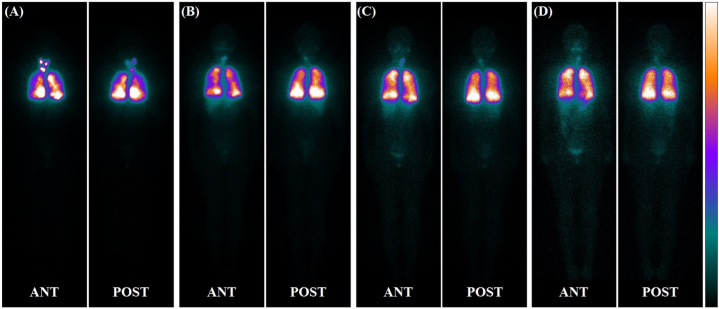
Iodine-131 treatment for DTC pulmonary metastases achieved SIR (the Rx-WBS imaging showed diffuse distribution) A 19-year-old female presented with follicular carcinoma of the thyroid gland in both lobes. Chest CT: The maximum diameter of the pulmonary metastases was 0.82 cm. Four times ^131^I treatments at a activity of 5.55GBq/time. Whole body anterior (ANT) and posterior (POST) plane imaging after ^131^I treatment. Imaging after initial ^131^I treatment (A): ^131^I uptake in the thyroid bed and pulmonary metastases showing diffuse ^131^I uptake. Imaging after the 2nd_4th treatment(B,C, and D): Faint uptake in the thyroid bed; pulmonary metastases showed diffuse ^131^I uptake, and the degree of ^131^I concentration was gradually reduced; TgAb value negative; sTg value was 605.5 ng/mL, TSH-suppressed Tg values were 47.06/33.80/21.57 and 18.52 ng/mL after 1st-4th ^131^I treatment, respectively.

A total of 26 (26/75, 34.7 %), 7 (7/75, 9.3 %), 38 (38/75, 50.7 %) and 4 (4/75, 5.3 %) patients achieved ER, BIR, SIR and IDR, respectively. Twenty-five (25/26, 96.2 %) patients achieved ER after two rounds of ^131^I treatments. The minimum cumulative activity of ^131^I administered was 9.25 GBq, and the maximum was 35.15 GBq. Fig. 1, Fig. 2 show the Rx-WBS and SPECT/CT images before and after treatment, respectively.

### 3.3 Clinical factors affecting ER

1) Univariate analysis: sTg value and Rx-WBS imaging type of initial ^131^I treatment were the factors affecting ER (z-value: −5.099, P < 0.001; χ^2^ value:10.457, P = 0.001). Since the p-value for metastatic nodule size was 0.054, it was included in the Binary Logistic regression analysis to avoid missing influencing factors.
2) Binary logistic analysis: Patients with lower sTg values, micronodular metastases, and focal distribution were more likely to achieve ER (odds ratio [OR]:0.998, 0.349, and 0.113, respectively; p < 0.001) (Table 1).

### 3.4 ROC curve analysis of sTg values predicting achieved ER

**Fig. 3:**
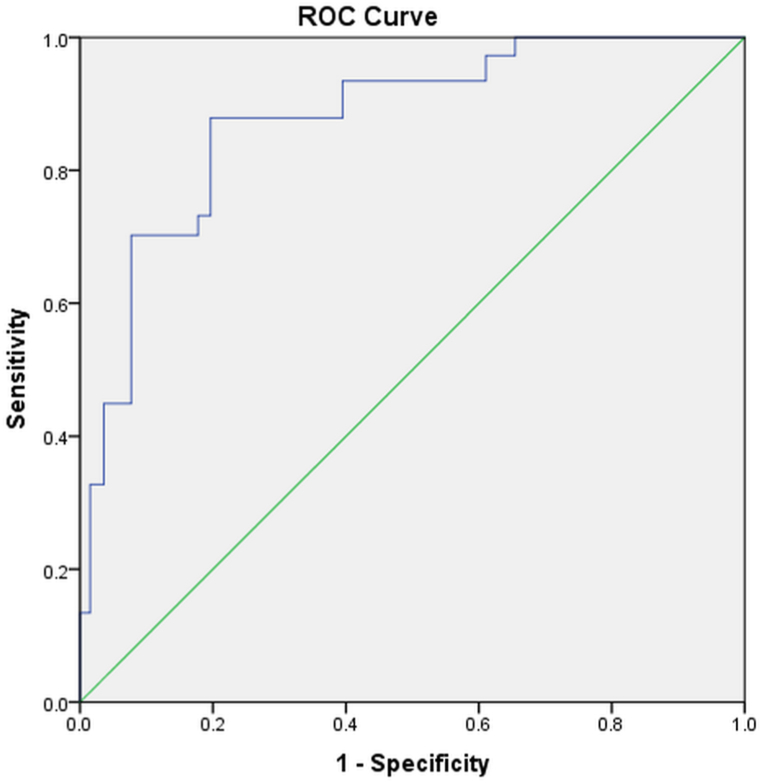
Receiver operator characteristic curve (ROC) for sTg value predicting ER for ^131^I treatment in ^131^I-avid DTC pulmonary metastases: AUC = 0.876, P < 0.001, the optimal threshold was 26.84 ng/mL, yielding a sensitivity of 87.9 % and specificity of 80.3 %.

The area under the ROC curve (AUC) for sTg value before initial ^131^I treatment for predicting ER was 0.876. The optimal threshold was 26.84 ng/mL, and the sensitivity and specificity for predicting ER were 87.9 % and 80.3 %, respectively. With the combination of sTg values, metastatic nodule size and Rx-WBS imaging type, the AUC was 0.883 (P = 0.000) (Fig. 3).

**Table 1:** Analysis of the factors influencing ER in 75 DTC patients with exclusively^131^I-avid pulmonary metastases.

Clinical date		ER	BIR + SIR + IDR	Univariate analysis and P value	Binary logistic regression analysis	OR	95%CI
age(year)	$\bar{x}$ ±*s*	47.0 ± 12.4	47.8 ± 14.0	1.587^a^; 0.113			
sTg	M(P25,P75)	11.3(2.8,34.1)	293(64.8,1688.5)	−5.099^b^; <0.001	<0.001	0.998	0.998–0.998
Stage (TNM)	Ⅰ+Ⅱ	22	39	0.279^d^; 0.598			
	Ⅲ+Ⅳ	4	10				
gender	male	5	16	0.921^c^; 0.337			
	female	21	33				
pathology type	PTC	21	42	0.309^c^; 0.578			
	FTC	5	7				
metastatic nodule size	≥1 cm	3	16	3.950^d^; 0.054	<0.001	0.349	0.273–0.444
	<1 cm	23	33				
Rx-WBS imaging type	focal	25	30	10.457^d^; 0.001	<0.001	0.113	0.083–0.154
	diffuse	1	19				

Note: a is the t value; b is the Mann-Whitney U rank sum test z value; c is the χ ^2^ value and d is the Fisher's test χ ^2^ value.

## 4 Discussion

According to the definition of radioiodine-refractory differentiated thyroid cancer (RAIR-DTC) [17], patients with non-131I-avid metastatic lesions fall under the RAIR-DTC category, and the potential benefits from ^131^I treatment are minimal, if any [18]. We reviewed and analyzed 109 patients with exclusively pulmonary metastasis from DTC. Based on classification using Rx-WBS and SPECT-CT imaging of pulmonary metastases, 28 patients with non-^131^I-avid metastatic lesions were considered as RAIR-DTC (22 with non-^131^I-avid metastatic lesions, 6 with partial ^131^I-avid metastatic lesions and partial non-^131^I-avid), and excluded. Pulmonary metastases were identified on the 2nd Rx-WBS imaging but not on initial Rx-WBS imaging in 6 patients. It may be affected by considerable residual thyroid tissue. These 6 patients with ^131^I-avid pulmonary metastases were not suitable for post-treatment status evaluation of RTC in the 2015 ATA guidelines and were also considered as RAIR-DTC, and subsequently excluded. Therefore, 75 patients with exclusively ^131^I-avid pulmonary metastases were selected for ^131^I treatment efficacy analysis.

Previous research on ^131^ I treatment for DTC distant metastases has mainly focused on activity selection, efficacy evaluation, efficacy influencing factors, the factors related to disease progression, and targeted drug therapy [19,20]. Our study investigated the clinically relevant factors affecting ER in DTC with ^131^I-avid pulmonary metastases patients. The 75 patients with exclusively ^131^I-avid pulmonary metastases enrolled in this study received ^131^I therapy, and 26 patients achieved ER, with a complete remission rate of 34.7 % (26/75). Previous studies have shown that the effective rate of ^131^I treatment for pulmonary metastases is 50–90 %, and that complete remission rate can reach 20–24.2 % [21,22]. Our study had a higher ER rate which can be explained by the inclusion criteria, patients with extrapulmonary distant metastases were excluded in our study. Compared to our research, previous studies included patients with extrapulmonary distant metastases, and these studies suggested that extrapulmonary metastases was an important predictor of poor outcome despite thyroid surgery and RIT [23].

According to the characteristics of radiotherapy, its efficacy of RAI therapy is related to the mean radiation dose delivered to neoplastic foci and also to the radiosensitivity of tumor tissue [24]. The clinical factors affecting the ER were investigated, including gender, age at diagnosis, pathological type, stages(TNM), sTg value, metastatic nodule size, and Rx-WBS imaging type. Univariate and binary logistic regression analyses revealed that the sTg value, metastatic nodule size， and the type of Rx-WBS imaging were independent factors influencing the ER. Previous studies have shown that age is an independent factor affecting efficacy and prognosis [25]. This study was analyzed based on whether ER was achieved in patients with exclusively ^131^I-avid pulmonary metastases. The one-factor measurement data *t*-test (t = 1.587; p = 0.113) did not reveal a significant difference in age between the two groups. Our study showed that age was significantly different between the ^131^I-avid and non-^131^I-avid groups, and older patients had a higher proportion in the non-^131^I-avid group.

This study incorporated the type of radioactivity distribution in Rx-WBS imaging into the analysis of the clinical factors affecting the ER. Univariate and Binary Logistic regression analyses suggested that Rx-WBS imaging type was an independent factor influencing ER, suggesting that patients with a focal distribution on Rx-WBS imaging were more likely to achieve ER. The following relevant factors could be considered: 1) the number of metastases with focal distribution was relatively fewer than with diffuse distribution; 2) diffuse distribution consisted mainly of diffuse miliary-like lesions, and there may be some small non-^131^I-avid metastatic lesions (Rx-WBS and SPECT/CT could not distinguish); 3) focal distribution may have a stronger effect of “cross-fire” to increase the radiation dose of the lesions. Previous studies have suggested that diffuse distribution is more common in children and young adults, and the proportion of patients achieving ER is 16 % [26]. Diffuse metastases may have a unique molecular pathological mechanism that is distinct from that of focal metastases and should be further explored.

Patients were categorized into groups with based on macronodular metastase and micronodular metastases. The analysis indicated that the size of the pulmonary metastases was an independent factor affecting the ER in the patients. Patients with micronodular metastases were more likely to achieve ER. Previous studies have established a significant correlation between efficacy and tumour size. The micronodular pattern of pulmonary metastases was invariably related to good ^131^I uptake, whereas macronodular metastases frequently showed poor ^131^I uptake [27]. For lung metastases with a volume of ≤0.8 mL (11.5 mm diameter), the average absorbed dose was 10–85 Gy, and the effective treatment rate reached 100 % [28]. In this study, 7 patients with negative CT imaging that are ^131^I uptake on ^131^I-WBS, were identified as ^131^I-avid pulmonary metastases. The size of metastatic lesions<2 mm were generally not seen on anatomic imaging, and defined as fine miliaric. Out of the 7 patients, 5 achieved ER. The highest ER rate was consistent with the findings of previous studies [29,30].

As a specific tumour marker for DTC, Tg has irreplaceable advantages in terms of sensitivity, specificity and practicality in assisting the diagnosis and efficacy evaluation [[31], [32], [33]]. Studies have also shown that the Tg value is also a reliable indicator for predicting disease-free remission and disease-related death [34,35]. Serum Tg value was reliable when TgAb was negative; otherwise, serum Tg levels could have been falsely lowered [36]. This study confirmed that sTg level before initial ^131^I treatment was an independent factor influencing patient ER, and that patients with lower sTg levels could achieve ER more easily.

Further ROC curve analysis of sTg value that predicted ER revealed that the area under the curve was 0.876, and the threshold was 26.84 ng/mL. Patients were more likely to achieve ER when sTg value before initial ^131^I treatment was lower than 26.84 ng/mL. Three patients with significantly high sTg values (3314 ng/mL, 698.81 ng/mL and 393.50 ng/mL) achieved ER after ^131^I treatment. Of the three patients, two had a maximum diameter of 2.34 cm and 1.73 cm, respectively. Clinical data showed that FT3 values were normal in one patient and slightly lower than normal in another patient before initial ^131^I treatment. Pulmonary metastases are thought to secrete thyroid hormone, leading to better therapeutic effects. The Rx-WBS imaging showed focal distribution in all 3 patients.

Our study has several limitations. First, this was a retrospective study. Some clinical data were missing, which might have led to a bias in data selection. Second, insufficient treatment may have occurred in some cases, resulting in deviations in the statistical data. Third, during the diagnosis of RAIR-DTC (with non-^131^I-avid metastatic lesions), histopathological confirmation was not always feasible, particularly for patients with partial ^131^I-avid metastatic lesions and partial non-^131^I-avid.

## 5 Conclusion

In conclusion, ^131^I treatment is effective for ^131^I-avid pulmonary metastases of DTC. Some patients who underwent ^131^I treatment achieved ER. Most patients with ER were obtained after two rounds of ^131^I treatments. Patients with sTg value before initial ^131^I treatment lower than 26.84 ng/mL, micronodular metastasis and focal distribution on Rx-WBS imaging were more likely to achieve ER.

## Ethic statement

This study was approved by Ethics Committee of Henan Provincial People's Hospital (People's Hospital of Zhengzhou University). The study was conducted according to established ethical guidelines and written informed consent obtained from all patients.

## Funding statement

This work was supported by the fund program of the Henan Key Laboratory of Molecular Nuclear Medicine and Translational Medicine (grant no.2020-27-4) and the 10.13039/501100006407Natural Science Foundation of Henan Province (grant no. 232300421174).

## Data availability statement

The data will be made available from the corresponding author upon request.

## CRediT authorship contribution statement

**Xin-Yu Wu:** Conceptualization, Data curation, Formal analysis, Investigation, Methodology, Visualization, Writing – original draft. **Bo Li:** Data curation, Formal analysis, Writing – original draft. **Jie Zhang:** Data curation, Formal analysis, Resources. **Li-Li Duan:** Data curation, Formal analysis, Resources. **Bing-Xin Hu:** Data curation, Formal analysis, Resources. **Yong-Ju Gao:** Conceptualization, Funding acquisition, Supervision, Writing – review & editing.

## Declaration of competing interest

The authors declare that they have no known competing financial interests or personal relationships that could have appeared to influence the work reported in this paper.
